# New Evidence-Based Treatment Approach in Behçet's Disease

**DOI:** 10.1155/2012/871019

**Published:** 2011-10-05

**Authors:** Erkan Alpsoy

**Affiliations:** Department of Dermatology and Venerology, Akdeniz University School of Medicine, 07059 Antalya, Turkey

## Abstract

Behçet's disease (BD) is a chronic, relapsing, and debilitating systemic vasculitis of unknown aetiology with the clinical features of mucocutaneous lesions, ocular, vascular, articular, neurologic, gastrointestinal, urogenital, and pulmonary involvement. The disease is much more frequent along the ancient “Silk Route” extending from Eastern Asia to the Mediterranean basin, compared with Western countries. The disease usually starts around the third or fourth decade of life. Male sex and a younger age of onset are associated with more severe disease. Although the treatment has become much more effective in recent years, BD is still associated with severe morbidity and considerable mortality. The main aim of the treatment should be the prevention of irreversible organ damage. Therefore, close monitoring, early, and appropriate treatment is mandatory to reduce morbidity and mortality. The treatment is mainly based on the suppression of inflammatory attacks of the disease using immunomodulatory and immunosuppressive agents. In this paper, current state of knowledge regarding the therapeutic approaches is outlined. To provide a rational framework for selecting the appropriate therapy along the various treatment choices, a stepwise, symptom-based, evidence-based algorithmic approach was developed.

## 1. Introduction

Behçet's disease (BD) is a chronic, relapsing, and debilitating systemic vasculitis of unknown aetiology with the clinical features of mucocutaneous lesions, ocular, vascular, articular, neurologic, gastrointestinal, urogenital, and pulmonary involvement [1]. BD usually starts around the third or fourth decade of life. Recent epidemiologic surveys [2–4] suggest that sex distribution is roughly equal. The disease is particularly prevalent in “Silk Route” populations but has global distribution. The pravelance of the disease is 14–20 per 100 000 along the Silk route [5]. Turkey has the highest prevalence. Azizlerli et al. from Istanbul reported the prevalence of the disease to be nearly 1/250 of the population aged 12 or older [2]. BD is rarely seen in western countries. The pravelance of the disease in England is less than 1/100 000 [5, 6]. This marked geographic variation of BD can be explained by the genetic basis of the disease and/or environmental triggers. The diagnosis is based on clinical criteria, as there is no pathognomonic test. Although several immunological abnormalities have been demonstrated, the exact mechanism of the inflammatory changes occurring remains to be elucidated. The most probable hypothesis is that of an inflammatory reaction set off by infectious agents such as herpes simplex virus 1 or *Streptococcus* spp. or by an autoantigen such as heat shock proteins in genetically predisposed individuals [7–9]. 

Mucocutaneous lesions figure prominently in the presentation and diagnosis and may be considered the hallmarks of BD. Oral ulcers (OUs), genital ulcers (GUs), and cutaneous lesions together with ocular lesions and arthropathy are the most frequent features of the disease in all countries. Mucocutaneous lesions often precede other manifestations. Therefore, their recognition may permit earlier diagnosis and treatment, with salutary results [5]. OUs are characterized by recurrent and painful ulcerations of the oral mucosa. They are identical to aphthae in appearance, but they tend to be more frequent and multiple. The most common sites are the mucous membranes of the lips, buccal mucosa, tongue, and soft palate. GUs are similar in appearance and course to OUs, but may not recur as often. They are usually deeper than the OUs and have a scarring tendency. The labia is the most frequently involved site in females and scrotum in males. Relapsing bipolar oral and genital ulcers are strongly evocative of BD [5]. Papulopustular lesions (PPLs) are sterile, folliculitis, or acne-like lesions on erythematous base which appear as a papule and in the course of a 24–48 hours become pustule. Trunk and the lower limbs are the most common locations [10]. Erythema nodosum (EN) is mostly seen in females and occur in about one-third of all patients. They have a typical clinical presentation with bilateral, pretibial, painful, and hot erythematous nodules. Other cutaneous lesions such as Sweet's syndrome-like, pyoderma gangrenosum-like, erythema multiforme-like lesions, extragenital ulcers, and palpable purpura can be seen during the course of the disease [1, 5, 11]. The skin pathergy test is a nonspecific skin hyperreactivity, induced by needle prick. The test positivity is defined as the development of a papule or pustule at the needle-prick site at 48 h. It is more strongly positive among males. Test positivity varies between geographic areas and has been reported to be high especially in Japan and the Mediterranean Sea countries (50–70%) [5].

Ocular involvement is a serious complication of BD and is characterized by repeated, explosive inflammatory attacks that may lead to visual loss in almost 15% of eyes. Panuveitis is the most frequent ocular lesion in BD. Anterior uveitis, posterior uveitis, and retinal vasculitis are the other main ocular manifestations. They are bilateral in most of the patients [12]. Articular involvement is characterized by nonerosive and nondeforming arthritis which often presents with monoarticular pattern, although asymmetrical polyarthritis can occur. The articular involvement is usually transient in nature with episodes lasting from a few days to weeks [13]. The disease is a systemic vasculitis affecting arteries and veins of various sizes. Venous system is the major affected site, and subcutaneous thrombophlebitis is, indeed, the most frequent type of venous involvement. Thromboses of the inferior vena cava and superior vena cava, dural sinuses, and Budd-Chiari syndrome can also be seen and are associated with poor prognosis. Pulmonary arterial aneurysm is rare; however, it is important cause of mortality [14]. Neurological involvement is relatively rare, but one of the most serious complications of the disease due to its grave prognosis. Parenchymal involvement including brainstem involvement, hemispheric manifestations, spinal cord lesions, and meningoencephalitis is seen in the majority of patients (%80). Dural sinus thrombi presenting with headaches and papilledema appear in 20% of patients with neurological involvement and have a more bening course [15]. Gastrointestinal involvement is characterized by aphthous-like mucosal ulcers occuring predominantly in the iliocaecal region, although it can occur throughout the gastrointestinal tract [16]. 

BD runs a chronic course with unpredictable exacerbations and remissions. In a recent multicenter study [17], we aimed retrospectively to determine the occurrence of the symptoms in chronologic order. We also evaluated the influence of the treatment and followup on the clinical severity and tried to obtain the factors determining the severe organ involvement in 661 patients. OUs were the most common manifestation (100%) followed by GUs (85.3%), PPLs (55.4%), EN (44.2%), skin pathergy reaction (37.8%), and articular (33.4%) and ocular involvement (29.2%). OUs were the most common onset manifestation (88.7%) which was followed by GUs (14.2%), EN (5.7%), and ocular involvement (4.2%). The duration between the onset symptom and the fulfilment of diagnostic criteria was calculated to be 4.3 ± 5.7 years. The frequency of ocular involvement and GUs was significantly higher in patients whose disease onset was less than 40 years. GUs, ocular involvement, PPLs, thrombophlebitis, and skin pathergy reaction were found to be significantly higher in males. The clinical severity of the disease showed a significant increase in noncompliant treatment group compared with compliant group with the passage of time. Our study showed that mucocutaneous lesions are the hallmarks of the disease, and especially OUs precede other manifestations. Male sex and a younger age of onset are associated with more severe disease.

Each or any combination of mucocutaneous, articular, and ocular symptoms of the disease may have significant pain or loss in function, or both. Besides considerable morbidity, the disease confers an increased mortality, mainly due to large vessel (especially pulmonary arterial) and neurologic involvement as well as bowel perforation. In general, mortality ratios as well as mucocutaneous and articular manifestations tend to decrease significantly with the passage of time. Both the onset of eye disease and its greatest damage are usually within the first few years of disease onset. A recent study [14] has shown that neurologic and large vessel involvements are exceptions, and they can have their onset late (5–10 years) during the disease course. In our multicenter study [17], in addition to these involvements, gastrointestinal involvement was also found to be a late manifestation of the disease. Therefore, all these results stress the importance of vigilance in long-term surveillance of patients with BD. Close monitoring and appropriate treatments are mandatory to decrease the morbidity and mortality of the disease since the disease shows a continuous activity.

## 2. Treatment

Treatment of the disease has become much more effective in recent years because of advances in understanding the pathogenesis the underlying disease and availability of a wide spectrum of therapeutic agents. Although several effective treatments currently exist, none of them result in a cure of the disease and some are associated with significant side effects. The choice of treatment is generally based on the clinical presentation and the site affected. However, the main aim of the treatment should be the prevention of irreversible organ damage, especially, during the early, active phase of the disease. Close monitoring and appropriate treatment may control and change the course of the disease. It is wise to remember that especially male patients and those with early onset disease are associated with more severe presentations including major vessel disease, ocular, gastrointestinal, and neurological involvement and, therefore, require more aggressive treatment [18].

This paper overviews the current state of knowledge regarding the therapeutic approaches for BD. Based on the mainly controlled studies and personal experience in clinical practice and basic research in this field, a stepwise, symptom-based, evidence-based algorithmic approach for the management of BD was proposed. This approach might enable clinicians to rationalize and further increase the selection of the most appropriate therapy among numerous treatment options [18].

### 2.1. Topical Treatment

The majority of experience in the treatment of OUs comes from the studies performed in patients with recurrent aphthous stomatitis (RAS). As we mentioned before, OUs of BD are identical to RAS in appearance. Therefore, therapeutic remedies related with RAS, to some extent, can be applied to OUs of BD. 

Although controlled studies are still lacking, the efficacy of topical *corticosteroids* is indisputable based on their favorable and widespread use. Topical corticosteroids suppress the inflammation associated with the formation of aphthae, and they are effective on both OUs and GUs especially when they are used in the early stage of these lesions. They reduce the pain severity and healing duration. Triamcinolone acetonide as cream 0.1% in Orabase or spray, prednisolone tablets in 20 mL water as rinse four times daily like those of dexamethasone elixir (0,5 mg/5 mL) can be used for OUs. Potent corticosteroid creams alone or in conjunction with antiseptics are also effective in GUs. Major OUs or GUs can be treated by intralesional triamcinolone, 5–10 mg/mL. Topically applied corticosteroid eye drops may also be used in mild attacks of anterior and intermediate uveitis together with mydriatics or cycloplegic agents [19]. *Antimicrobial agents* including antiseptic agents and antibiotics are used to control microbial load [1]. Two controlled studies with antiseptic agents, listerine mouth rinse [20], and chlorhexidine gel [21] in RAS noted the effectiveness on the pain severity and duration. Triclosan, a broad-spectrum antibacterial agent has been shown to reduce the number of aphthous ulcers in a double-blind cross-over study in RAS patients [22]. Antibiotics, especially tetracycline has been widely used in OUs of BD for years. Tetracycline mouthwash (250 mg capsules dissolved in 5 mL of water or flavored syrup and held in the mouth for about 2 minutes before swallowing four times daily) decrease pain severity and duration of OUs. A double-blind trial of tetracycline suspension showed significant reductions in ulcer duration, size, and pain in RAS patients [23]. A recent study [24] assessed 0.2 percent minocycline and 0.25 percent tetracycline aqueous solution mouthwash in patients with RAS in a clinical randomized crossover trial. Minocycline mouthwashes as compared to topical tetracycline rinses resulted in significantly improved pain control, by reducing the severity and duration of pain. Cephalexin [25] and penicillin G [26] have also been reported to be effective antibiotics. *Sucralfate* (1 g/5 mL), 4 times daily, for 3-month duration as mouthwash, decreases significantly the frequency, healing time, and pain of OUs and healing time and pain of GUs. The effectiveness of the sucralfate on the OUs frequency and healing time continue during the posttreatment period in decreasing order [27]. This compound binds to ulcerated tissue and forms a barrier and augments ulcer healing. Recent controlled studies suggest that *pimecrolimus,* a topical immunomodulator, twice a day seems to be safe and efficient in the treatment of genital ulcers, by accelerating the healing process and shortening the pain duration [28, 29]. *Amlexanox* accelerate the healing and decreases the pain severity of oral ulcers. It has anti-inflammatory and antiallergic activities [30, 31]. Amlexanox is used in oral paste (5%) 4 times daily (after meals and at bedtime) for 4–10 days. *Anti-inflammatory agents* (benzydamine, diclofenac), *anaesthetics* (lidocaine 2–5%, mepivacaine 1.5%, tetracaine 0.5–1% gels, or mucosal ointments), and *silver nitrate*, in general reduce the pain severity of aphthous lesions [5, 32, 33]. Recently, beneficial effects of *colony stimulating factor* on the healing duration and pain severity of OUs and GUs have been reported by our group [34]. 

In addition to the above-mentioned treatment approaches to OUs, patients should be advised to maintain good daily oral hygiene [35]. These patients should avoid irritating agents such as acid, crusty, hard, spicy, or salty nutrients and alcoholic beverages. EN is treated topically like classic EN. *Wet dressings* such as aluminium acetate 3–5% (Burow's solution) can be applied in early stage of these lesions. This approach is also helpful for the treatment of superficial thrombophlebitis. All therapy should be combined with rest in bed.

### 2.2. Systemic Treatment

#### 2.2.1. Corticosteroids

Corticosteroids have been widely used almost for all lesions of the disease. The compound is an effective choice especially in mucocutaneous lesions, acute uveitis, and neurologic disease. They can be given as monotherapy or in combination with other drugs such as colchicine, interferon (IFN)-*α*, cyclosporine, or azathioprine. However, in a recent randomized, placebo-controlled study of 86 patients who had active mucocutaneous lesions without eye and major organ involvement, low dose depot steroid (40 mg of methylprednisolone acetate every 3 weeks) was only found to be helpful in controlling EN, especially among females [36]. However, this result does not mean the compound is not effective in daily and/or higher doses. On the other hand, the well-known side effect profile limits their long-term use, and more corticosteroids do not improve the long-term outcome.

#### 2.2.2. Colchicine

Colchicine inhibits the enhanced chemotactic activity of neutrophils. Promising results with colchicine (0.5–2 mg/d p.o.) have been reported especially in mucocutaneous and articular findings. The first placebo-controlled study suggested that the drug is effective only for EN and arthralgia [37]. Yurdakul et al., in a randomized placebo-controlled study [38], revisited the issue and have shown that colchicine reduces the occurrence of GUs, EN, and arthritis among women and the occurence of arthritis among men. Although oligozoospermia, amenorrhea, or dysmenorrhea, malaise, hair loss, gastrointestinal complaints (nausea, vomiting, diarrhea), and hematologic side effects are recorded as the main adverse effects of colchicine, Yurdakul et al. [38] reported no significant difference between the groups.

Recently, Davatchi et al. [39], in a large cohort of BD patients (169 patients without major organ involvement), reevaluated the efficacy of colchicine. In this randomized, double-blind, controlled crossover trial, the overall disease activity index and OUs, GUs, PPLs, and EN improved significantly with colchicine. There was not any significant difference between the results for males and females.

#### 2.2.3. Benzathine Penicillin

Calguneri et al. [40] have found that the combined use of colchicine and benzathine penicillin (1.2 MU/3 weeks) treatment more effective than colchicine alone. Combined treatment was effective in reducing frequency and duration of OUs and EN and the frequency of GUs. Combined treatment also significantly reduced the number of arthritis episodes and prolonged the duration of episode-free time compared with the colchicine-alone group. Recently Al-Waiz et al. [41] showed that combined use of colchicine (1 g/d) and benzathine penicillin (1.2 MU/m) is more effective in decreasing clinical manifestation index, the numerical sum of the clinical features, than in either drug alone.

#### 2.2.4. Rebamipide

In a double-blind, placebo-controlled study of 35 BD patients, having as the main symptom OUs, Matsuda et al. [42] used rebamipide (300 mg/day) for 3 to 6 months. They reported that the rate of moderate or marked improvement in OUs count and pain was 36% in the placebo group and 65% in the drug group. Authors concluded that rebamipide may be useful in the treatment and prevention of recurrences of OUs. No significant adverse effect has been reported.

#### 2.2.5. Zinc Sulfate

In a recent controlled study of 32 patients, Sharquie et al. [43] evaluated the efficacy of zinc sulfate in a double-blind, crossover study and reported an improvement in the clinical manifestations index of mucocutaneous lesions without any side effect. 

#### 2.2.6. Dapsone

Dapsone also inhibits the enhanced chemotactic activity of neutrophils and can be used as an alternative compound to colchicine. In a double-blind, crossover study of 20 patients, Sharquie et al. reported significant reductions in the number, duration, and frequency of OUs and number of GUs in dapsone-treated patients. This compound also showed a significant decrease in the frequency of EN and PPLs. Arthritis and epididymitis were also significantly supressed by dapsone, but the effect of the compound on arthralgia failed to reach the level of statistical significance [44]. Hemolytic anemia and methemoglobinemia, which can be severe in patients with glucose-6-phosphate dehydrogenase deficiency, are the main side effects, which may significantly limit their use.

Despite the encouraging results of the last three studies, a limited number of patients included, and a relatively short follow-up periods were the main limitations.

#### 2.2.7. Thalidomide

The drug selectively inhibits TNF-*α* synthesis. In a randomised, double blind, placebo-controlled study with 63 patients, a remission of OUs, GUs, and PPLs was detected in 22% of the patients over 8 weeks. During the 6-month treatment 30% of the patients remained free of lesions. Thalidomide therapy, however, was associated with exacerbation of EN [45]. In addition, the effects of the drug are temporary, and discontinuation of the treatment results in recurrence of the OUs and GUs. The effectiveness of the thalidomide is lost about 20 days after discontinuation of the drug. Neurological side effects and teratogenic risc of thalidomide limit the clinical application. 

#### 2.2.8. Azathioprine

Azathioprine, an important disease-modifying compound, shows an anti-inflammatory effect by suppressing both cellular and humoral immune responses. In a randomised, double-blind, placebo-controlled study [46] of 73 patients, azathioprine has been found to be an effective choice in OUs and GUs besides ocular inflammation and arthritis. Azathioprine was significantly better than placebo in preventing the development of new eye disease. Therefore, the authors concluded that the drug can be used prophylactically to prevent the eye involvement in young, male patients presenting with severe mucocutaneous lesions. Myelotoxicity, gastrointestinal complaints, immunosuppression, opportunistic infections, and hepatotoxicity are the main side effects.

#### 2.2.9. Cyclophosphamide

Cyclophosphamide is the fast-acting alkylating agent. It has been found as a beneficial therapeutic agent for eye disease and systemic vasculitis (neurologic involvement and arterial aneurysms). In a double-blind crossover study [47], it has been shown that the combination of cyclophosphamide and corticosteroid therapy is superior to corticosteroid therapy alone in eye involvement. Myelosuppression, pulmonary fibrosis, renal toxicity, hemorrhagic cystitis, infertility, malignancy, and alopecia are the major adverse effects of cyclophosphamide. Due to the severe toxicity, cyclophosphamide should be selected in cases with clinically significant disease who are refractory to other agents.

#### 2.2.10. Cyclosporin A

Cyclosporin A (CyA) is an immunosuppressant agent which selectively inhibits T lymphocytes. The drug is capable of markedly ameliorating uveitis as well as mucocutaneous lesions. CyA is still one of the most effective agents for the treatment of uveitis which reduces the frequency of ocular exacerbations and improves visual acuity. In a conrolled study of 96 patients with recurrent uveitis, CyA (10 mg/kg/d) has been shown to be superior to colchicine (1 mg/d) in decreasing frequency and severity of ocular attacks [48]. In the study of BenEzra et al. [49], CsA was more effective than conventional therapy (prednisolone, chlorambucil) in decreasing the active ocular inflammatory processes and arresting the deterioration of visual acuity. However, conventional therapy was superior to CsA in controlling OUs, GUs, and arthritis. Elidan et al. [50] reported that CyA is significantly better than conventional therapy (prednisolone, chlorambucil) at improving hearing loss. Five of 20 Behçet patients under CyA therapy demonstrated improvement in their hearing loss. In a comparative study [51], a significant improvement in visual acuity during the first 6 months in CyA (5 mg/kg/d) group compared with cyclophosphamide (1000 mg/mo) was observed. However, this favorable effect of CyA was not sustained in the followup of patients up to 24 months. In another controlled trial [52], 26 patients treated with CyA with a dose of 5 mg/kg/d have been compared with 50 patients receiving conventional therapy, systemic corticosteroid alone or combined with azathioprine. CyA treatment was found to be more effective in reducing OUs, GUs, cutaneous lesions, thrombophlebitis as well as articular symptoms and neurologic symptoms. Therefore, CyA is also an effective alternative for mucocutaneous lesions; however, it should be reserved for the most severe cases because of its significant long-term adverse effects such as renal failure, hypertension, neurologic toxicity, and hirsutism. It is wise to remember that neurological manifestations occur more frequently in BD patients under CyA treatment [53].

#### 2.2.11. Interferon-*α*

In recent years, the increasing evidence suggests that interferon (IFN)-*α* is an effective alternative in the treatment of BD. The mode of action of IFN-*α* in BD is still unknown. However, their antiviral and immunomodulatory effects appear to be the possible mechanisms. In a randomised, double-blind, placebo-controlled study [54], we have shown that IFN-*α* 2a treatment is an effective alternative particularly for the management of mucocutaneous lesions, and its effect decreases gradually after the cessation of treatment. IFN-*α* 2a treatment decreased significantly the duration and pain of OUs and the frequency of GUs and PPLs. Although not significant, the mean frequency and duration of EN, thrombophlebitis, and articular symptoms also showed a decrease. Hamuryudan et al. [55], in their 48-week open, self-controlled trial, reported that IFN-alpha 2b significantly reduced the mean number of arthritis attacks. 

IFN-*α* has also been employed in cases of sight-threatening refractory uveitis with promising results. Kötter et al. in their open-label, prospective study [56] used IFN-*α* 2a in 50 patients at a dose of 6 million IU (MIU) daily, tapered according to a preset schedule. The authors concluded that IFN-*α* 2a is effective in ocular BD, leading to significant improvement of vision and complete remission of ocular vasculitis in the majority of the patients. Tugal-Tutkun et al. [57] evaluated the IFN-*α* treatment in 44 patients with uveitis unresponsive to conventional immunosuppressive therapy. Although the overall response rate was 91%, complete response rate (36.4%) was lower than that of the study of Kötter et al. In a newer study, Onal et al. [58] investigated the long-term efficacy and safety of low dose (3.0 MIU daily for 14 days, maintenance dose, 3 MIU 3 times per week for 24 months) therapy of IFN-*α* 2a in 37 patients with refractory Behçet panuveitis unresponsive to conventional immunosuppressive therapy. During maintenance therapy, IFN-*α* 2a controlled uveitis in 35 patients (95%). In 15 patients (41%), a maintenance dosage of 3 MIU 3 times per week controlled uveitis without any relapse. Remission rate after discontinuation of IFN-*α* 2a therapy was 76% by 3 months. Therapeutic response rate differences of ocular BD with IFN-*α* treatment among the respected studies might have been caused by the different patient populations studied and the different dose schedules. Nevertheless, taken together, IFN-*α* 2a seems to be able to control and achieve remission of uveitis in most patients with refractory ocular BD.

The primary side effects of IFN-*α* therapy are flulike symptoms (fever, chills, headache, fatigue, myalgia, etc.) that start a few hours after the initiation of the therapy and continue less than a day. We use oral acetaminophen (paracetamol) 1000 mg orally before injections and 500 mg after 6 hours during the first weeks of the therapy to decrease these side effects. Nausea, vomiting, anorexia, diarrhea, loss of weight, hematologic changes, transient raising of hepatic transaminases are seen less frequently. Psychiatric side effects and depression are limiting factors for use of IFN-*α*.

#### 2.2.12. Anti-TNF-*α* Agents

Several pieces of evidence indicate that TNF-*α* plays a critical role in the pathogenesis of BD, and so far, three anti-TNF-*α* compounds, infliximab, adalimumab, and etanercept, have shown favourable results on preliminary tests. Almost all trials reported encouraging results for recalcitrant mucocutaneous lesions besides ocular and gastrointestinal symptoms, arthritis, and cerebral vasculitis. Anti-TNF-*α* treatment suppresses almost all manifestations of the disease with an immediate and dramatic response. It also reduces the dosage of immunosuppressors. Therefore, when the disease is associated with vital organ involvement, especially in young male patients, anti-TNF-*α* agents can also be used because of their potential to improve the survival and prognosis.

The majority of current data related with infliximab comes from the uncontrolled, open studies, small case series, and case reports. The main 3 prospective studies performed by Sfikakis et al. [59], Ohno et al. [60], and Tugal-Tutkun et al. [61] concentrated on the eye disease and reported promising results. Besides these studies, many small case series and case reports suggest that patients with mucocutaneous lesions, gastrointestinal symptoms, arthritis and cerebral vasculitis exhibit rapid and good responses to infliximab [62]. Recently, adalimumab, a fully humanised anti-TNF-*α* antibody, has also been reported to be an effective alternative [63].

Melikoglu et al. [64] conducted the first controlled study of anti-TNF-*α* compound, etanercept. In a double-blind, placebo-controlled study of 40 male patients, authors reported that etanercept (25 mg twice a week for 4 week) is effective in suppressing most of the mucocutaneous lesions. The drug had a clear effect on OUs and nodular lesions, and the response was as early as the first week. There was a significant decrease in the mean numbers of OUs and nodular lesions as well as PPLs. 

A recent position paper concluded that infliximab is recommended as an add-on therapy for severe BD, refractory or intolerant to traditional immunosuppressive regimens. Moreover, a single infusion of infliximab (5 mg/kg) can be used as a first-line agent for sight-threatening, bilateral posterior eye segment inflammation, because the fast onset of response is critical to prevent fixed retinal lesions, and therefore, permanent visual loss. In case when ocular relapses are not controlled by azathioprine and/or cyclosporin, maintenance therapy with 5 mg/kg doses of infliximab every 6–8 weeks could be used for 2 years, provided no relapses occur between intervals [62].

However, the high cost, the need for injections, troublesome toxic side effects, and the inability to cure the disease are the main limitations for widespread acceptance of anti-TNF-*α* as a first-line choice for the management of BD. Optimal dosage and the long-term consequences are still important questions for anti-TNF-*α* agents to be answered. It still needs further controlled studies in large series. 

Adverse effects of anti-TNF-*α* agents include infection (sinusitis, pharyngitis, bronchitis, and urinary tract infections, reactivation of tuberculosis), autoimmune reactions (e.g., lupuslike syndrome), lymphoproliferative disorders, delayed hypersensitivity reactions, and neurologic, cardiac, and gastrointestinal symptoms.

#### 2.2.13. Rituximab

Rituximab is a chimeric monoclonal antibody against CD20, a B-cell differentiation marker. Recently, Davatchi et al. [65] used rituximab in their randomized single-blind controlled study. Twenty patients were randomized to a rituximab group (in two 1000 mg courses, 15-day interval) or cytotoxic combination therapy group. Patients with rituximab group were also given methotrexate (15 mg/weekly) with prednisolone (0.5 mg/kg per day). The cytotoxic combination therapy group received pulse cyclophosphamide (1000 mg/monthly), azathioprine (2–3 mg/kg per day), and prednisolone (0.5 mg/kg per day). The authors concluded that rituximab was efficient in severe ocular manifestations of BD, and total adjusted disease activity index improved significantly after 6 months with rituximab, but not with cytotoxic combination therapy group.

#### 2.2.14. Other Systemic Treatment Approaches

Several open studies of *methotrexate* (7.5–20 mg/1x week p.o. over 4 weeks) have reported the induction of an improvement of a severe mucocutaneous involvement [66] as well as neurological [67, 68] and ocular involvement [69]. Methotrexate is not recommended in pregnancy and lactation, and severe bone marrow depression, liver dysfunction, acute infections, renal insufficiency, and mucositis are important side effects of the drug. *Mycophenolate mofetil *(MMF) was found to be safe and effective in controlling cystoid macular oedema and in reducing the uveitis relapse rate in patients not responding to traditional immunosuppressants [70]. On the other hand, an open study reported no benefit in mucocutaneous disease [71]. MMF is generally well tolerated; the most common side effects involve gastrointestinal and genitourinary symptoms. Other reported less frequent adverse events include neurologic, cutaneous, cardiorespiratory, and metabolic reactions. Rarely, severe leukopenia has also been reported. *Autologous hematopoietic stem cell transplantation* has recently been reported as a successful treatment option for severe/refractory patients with intestinal [72], pulmonary [73], and neurologic [74] involvements. Open studies with *Pentoxifylline* reported good results on mucocutaneous symptoms. However, recurrences occurred in all patients after discontinuation of treatment. Pentoxifylline has also been described as alternative treatments for ocular lesions in few patients with BD [75, 76]. *Sulfasalazine* (2–4 gr/day) was reported to be an effective choice for the treatment of gastrointestinal involvement [77].

### 2.3. Surgical Treatment

Although various treatment modalities appear, surgical intervention often is indicated for arterial aneurysms. In patients with recurrent or massive hemoptysis, surgery may be necessary. Endovascular treatment for pseudoaneurysms due to BD seems to be an effective choice when the disease activity is strictly controlled with immunosuppressive therapy [78, 79]. In other serious consequences, such as gastrointestinal bowel perforation, enterocutaneous fistula formation, thrombotic obstruction in large-caliber vessels, cardiac involvement, and complications of eye involvement such as glaucoma, vitreous opacities, surgery may also be the only possible remedy [19, 80].

## 3. Evidenced-Based Algorithmic Treatment Approach in Behçet's Disease

Activity spectrum of systemic therapeutic agents on BD in randomized, controlled studies is summarized in Table 1. A stepwise, symptom-based, algorithmic approach, mainly based on controlled studies and our clinical experience in this field, is summarized below and in Tables 2–6.

### 3.1. Mucocutaneous Disease

Colchicine should be the first choice in the treatment of GUs and/or EN, especially in female patients [38]. If it is not effective or patient is male, colchicine can be combined with benzathine penicillin [40, 41]. In the presence of OUs with or without other mucocutaneous lesions, this combination should also be the starting point. 

Short-term corticosteroids in combination with other drugs such as colchicine can be used as alternatives in the treatment of acute attacks of mucocutaneous lesions [18]. Dapsone can also be used at this stage as an effective compound [44]. Patients with severe mucocutaneous disease or those who are unresponsive to the respected treatments can be treated with azathioprine [46]. Thalidomide [45] is an effective choice. However, because of potential side effects, it should be used cautiously in selected patients. It is wise to keep in mind that EN worsens during thalidomide treatment. 

Rebamipide [42], zinc sulfate [43], and Pentoxifylline [75] can be used as 3rd-line treatment choices. However, there is still a need for well-organised newer studies for these agents. In severe cases and/or unresponsive cases to the other treatments, methotrexate [66], cyclosporin [52], and biologicals such as IFN [54] and anti-TNF-*α* agents [62, 64] can be used to control the disease. 

Antimicrobial agents [20–26], sucralfate [27] and corticosteroids [19] especially in OUs, and pimecrolimus [28, 29] in GUs can be selected as 1st-line topical treatment choices. Anti-inflammatory agents, amlexanox, anaesthetics, and silver nitrate [30–33] are other alternatives for topical treatment of mucocutaneous lesions. However, it is very important to ensure for clinician that topical treatment, with a great possibility, has only local effects and should almost always be associated with systemic therapy.

Evidenced based algorithmic treatment approach for mucocutaneous Behçet's disease is summariezed in Table 2. 

### 3.2. Articular Disease

Evidenced-based algorithmic treatment approach for articular Behçet's disease is summariezed in Table 3. 

Colchicine should be the first choice [37, 38]. Additional use of benzathine penicillin or anti-inflammatory analgesics can be the next step [40, 41]. 

In unresponsive cases, azathioprine [46] can be an alternative. Low dose corticosteroids, and even intraarticular corticosteroid injections in monoarticular involvement, can also be used as 2nd-line treatment [77, 81]. 

Although controlled studies are still lacking, methotrexate and salazopyrine are used successfully in the clinical practice [77]. IFN-*α* [55] and anti-TNF-*α* agents [62] are the other alternatives.

### 3.3. Ocular Disease

Evidenced-based algorithmic treatment approach for ocular Behçet's disease is summarized in Table 4. 

Ocular involvement requires special attention and usually aggressive treatment since it has the highest morbidity. In mild uveitis such as anterior uveitis, topically applied corticosteroid eye drops together with mydriatics or cycloplegic agents can often control the disease [19]. Systemic corticosteroids should be the next step. It is wise to remember that systemic corticosteroids are also used in acute inflammatory ocular attacks of posterior uveitis, panuveitis, and retinal vasculitis [18]. Systemic corticosteroids should be used in brief courses for long term because of well-known side effect profile. Unresponsive cases, those with posterior uveitis, or those who develop chronic, steroid-dependent intraocular inflammation (given the deleterious effects of chronic steroid administration to the eye) require more aggressive treatment. Immunosuppressives such as azathioprine [46] and cyclosporine [48, 49, 51] are the main choices. Cyclosporine together with corticosteroids can be used effectively. Azathioprine and cyclosporine can also be combined in those patients whose eye disease is refractory to treatment [18]. 

As we mentioned before, IFN-*α* or anti-TNF-*α* treatments can be used in case when the immunosuppressives do not control the disease [82].

Methotrexate, MMF, cyclophosphamide, and rituximab can be used in selected patients as a 3rd-line therapy [47, 65, 69, 70]. 

In the most severe cases with retinal vasculitis or macular involvement, CyA or anti-TNF-*α* treatments can be combined with azathioprine and corticosteroids. Cyclophosphamide and IFN-*α* with or without corticosteroids are other alternatives for the treatment of severe eye disease [18].

### 3.4. Severe Disease

Although several promising therapies are evolving, the treatment of severe disease is not entirely satisfactory and treatment of those remains predominantly empirical. Severe disease has relatively lower incidence. Because of the limited number of patients enrolled in studies in this area statistical comparisons were usually not made. These factors make recommendation of individual treatments difficult for these involvements.

Evidenced-based algorithmic treatment approach of Behçet's disease with large vessel, neurologic, and gastrointestinal involvement is summarized in Tables 5–7.

#### 3.4.1. Large Vessel Involvements

In the presence of deep vein thromboses, azathioprine can be used. In severer cases with inferior vena cava or superior vena cava syndrome and Budd-Chiairi syndrome cyclophosphamide as monthly pulse treatment should be added to the treatment. It is unclear the effectiveness of additional use of antiplatelets or anticoagulation [18, 77, 81, 82].

In arterial involvement, corticosteroids together with cyclophosphamide are generally preferred to control the disease. Anticoagulation should not be given in the presence of pulmonary arterial aneurysm because of the danger of bleeding [77, 81, 82]. Anti-TNF-*α* agents, especially infliximab, can be alternative [18].

Surgery may be necessary in life-threatening conditions such as growing aneurysm, acute rupture [81].

#### 3.4.2. Neurologic Involvements

In parenchymal involvement, corticosteroids (100 mg/d or 1 gx 5 days as pulse treatment) should be the first choice. Azathioprine is usually combined with corticosteroids. In severe or unresponsive cases, cyclophosphamide can be given additionally [83]. Anti-TNF-*α* agents and IFN-*α* are other new effective alternative agents [19]. Methotrexate is another treatment alternative [67, 68].

In venous sinus thrombosis corticosteroids with or without immunosuppressives are the main treatment approaches. In this situation additional use of anticoagulation is also suggested [81, 83].

#### 3.4.3. Gastrointestinal Disease

Sulfasalazine and corticosteroids seem to be the 1st-line treatment options [77]. Azathioprine can be used effectively in unresposive cases. Anti-TNF-*α* treatments, especially infliximab, seem to be new and effective alternative. Surgery should be selected in those patients with perforation and intractable bleeding [18, 81]. 

In conclusion, treatment of BD has become much more effective in recent years. Due to recent advances in understanding the pathogenesis of the underlying disease and availability of a wide spectrum of therapeutic agents, alleviation of most symptoms, control of the disease, and, even, modification of the course of the disease are now possible.

## Figures and Tables

**Table 1:** Activity spectrum of systemic therapeutic agents on Behçet's disease in randomized, controlled studies.

Treatment	Dose	Indication and reference
Corticosteroids versus placebo	40 mg/every 3 w	Decrease the frequency of EN in women [36]
Colchicine versus placebo	1–2 mg/d	Decreases the frequency of EN and effective on arthralgia [37] Reduces the occurrence of GUs, EN, and arthritis in women and the occurrence of arthritis in men [38]
	1 mg/d	Decrease in overall disease activity index and significant improvement in OUs, GUs, PPLs, and EN [39]
Colchicine versus Colchicine + Benzathine penicillin	1–2 mg/d; 1.2 MU/3 w	Combined treatment more effective in reducing frequency of arthritic episodes, duration and frequency of OUs and EN, and the frequency of GUs [40]
Colchicine versus Benzathine penicillin versus Colchicine + Benzathine penicillin	1 mg/d; 1.2 MU/mo	Combined use of colchicine and benzathine penicillin treatment more effective than colchicine or penicillin alone [41]
Rebamipide versus placebo	300 mg/d	Reduces the number of OUs and pain [42]
Zinc sulfate versus placebo	300 mg/d	Significant improvement in the clinical manifestations index of mucocutaneous lesions [43]
Dapsone versus placebo	100 mg/d	Effective on the number, healing time and frequency of OUs, number of GUs, and frequency of EN and PPLs. Suppresses arthritis and epididymitis [44]
Thalidomide versus placebo	100–300 mg/d	Sustained remission of OUs, GUs, and PPLs [45]
Azathioprine versus placebo	2,5 mg/kg/d	Reduces the occurrence of OUs, GUs, arthritis, and ocular symptoms. Prevents the development of new eye disease [46]
Cyclophosphamide + Corticosteroids versus Corticosteroids	1 g/m^2^/mo	Combined treatment of CCP and corticosteroids more effective in eye disease than corticosteroids alone [47]
Cyclosporin A versus Colchicine	10 mg/kg/d	CyA more effective on the severity and frequency of OUs, GUs, and PPLs. Superior to colchicine in decreasing the frequency and severity of ocular attacks [48]
Cyclosporin A versus conventional treatments (prednisolon, chlorambucil)	10 mg/kg/d	CsA more effective than conventional therapy in ocular disease, however, conventional therapy superior to CyA in controlling OUs, GUs, and arthritis [49]
Cyclosporin A versus conventional treatments (prednisolon, chlorambucil)	10 mg/kg/d	Improvement of hearing loss in 25% of patients receiving CyA treatment [50]
Cyclosporin A versus Cyclophosphamide	5 mg/kg/d	A significant improvement in VA during the first 6 months in CyA group compared with CCP [51]
Cyclosporin A versus conventional treatments (prednisolon, Azathioprine)	5 mg/kg/d	CyA more effective than conventional therapy in OUs, GUs, cutaneous lesions, thrombophlebitis as well as articular and neurologic symptoms [52]
Interferon-*α* versus placebo	6 MU/d-3 x/w	Effective on pain and healing time of OUs and frequency of GUs and PPLs. Also helpful in decreasing frequency and duration of EN, TFB, and articular symptoms [54]
Etanercept versus placebo	25 mg/d-2 x/w	Reduces the occurrence of OUs, nodular skin lesions, and PPLs [64]
Rituximab versus cytotoxic combination therapy	2 1000-mg courses (15-day interval)	A significant improvement in total adjusted disease activity index in rituximab group [65]

d: day; EN: erythema nodosum; GUs: genital ulcers; Mo: month; OUs: oral ulcers; PPLs: papulopustular lesions; TFB: thrombophlebitis; VA: visual acuity; w: week.

**Table 2:** Summary of evidence-based algorithmic treatment for mucocutaneous Behçet's disease.

1st line	*Topical: Antimicrobial agents, Sucralfate, Corticosteroids, Pimecrolimus
	Systemic: Colchicine, Colchicine + Benzathine penicillin

2nd line	*Topical: Anti-inflammatory agents, Amlexanox
	Systemic: Corticosteroids, Dapsone, Azathioprine, Thalidomide

3rd line	*Topical: Anaesthetics, Silver nitrate
	Systemic: Zinc sulfate, Rebamipide, Pentoxifylline, Methotrexate, Cyclosporine-A, IFN-*α*, Anti-TNF-*α*

*Since the effectiveness of topical treatment is generally limited to the application area, it should almost always be associated with systemic therapy.

**Table 3:** Summary of evidence-based algorithmic treatment for articular Behçet's disease.

1st line	Colchicine, Colchicine + Benzathine penicillin, or anti-inflammatory analgesics
2nd line	Azathioprine, Corticosteroids
3rd line	Methotrexate, Salazopyrine, IFN-*α*, Anti-TNF-*α*

**Table 4:** Summary of evidence-based algorithmic treatment for ocular Behçet's disease.

1st line	*Topical: corticosteroids + mydriatics ± cycloplegic agents
	Systemic: Corticosteroids, Cyclosporine-A, Azathioprine
2nd line	IFN-*α*, Anti-TNF-*α*
3rd line	Methotrexate, Mycophenolate mofetil, Cyclophosphamide, Rituximab

*Topical treatment as a sole agent should be restricted to those who has mild uveitis (anterior uveitis).

**Table 5:** Summary of evidence-based algorithmic treatment for Vasculo-Behçet disease.

1st line	Corticosteroids, Azathioprine, Cyclophosphamide,
2nd line	Anti-TNF-*α*
3rd line	Anticoagulation, Antiplatelets

**Table 6:** Summary of evidence-based algorithmic therapy for Neuro-Behçet's disease.

1st line	Corticosteroids
2nd line	Azathioprine, cyclophosphamide, Anti-TNF-*α*, IFN-*α*
3rd line	Methotrexate, Anticoagulation

**Table 7:** Summary of evidence-based algorithmic therapy for gastrointestinal Behçet's disease.

1st line	Sulfasalazine, corticosteroids
2nd line	Azathioprine
3rd line	Anti-TNF-*α*
